# Experimental Investigation on Magnetic Abrasive Finishing for Internal Surfaces of Waveguides Produced by Selective Laser Melting

**DOI:** 10.3390/ma17071523

**Published:** 2024-03-27

**Authors:** Liaoyuan Wang, Yuli Sun, Zhongmin Xiao, Liming Yao, Jiale Guo, Shijie Kang, Weihao Mao, Dunwen Zuo

**Affiliations:** 1 College of Mechanical and Electrical Engineering, Nanjing University of Aeronautics and Astronautics, Nanjing 210016, China; n2308512h@e.ntu.edu.sg (L.W.); guojiale@nuaa.edu.cn (J.G.); kangshijie0322@nuaa.edu.cn (S.K.); maoweihao@nuaa.edu.cn (W.M.); imit505@nuaa.edu.cn (D.Z.); 2 School of Mechanical and Aerospace Engineering, Nanyang Technological University, Singapore 639798, Singapore; liming.yao@ntu.edu.sg; 3 School of Mechatronics Engineering, Harbin Institute of Technology, Harbin 150080, China; 4 Zhengzhou Research Institute, Harbin Institute of Technology, Zhengzhou 450000, China

**Keywords:** selective laser melting, AlSi10Mg metal powders, magnetic abrasive finishing, surface roughness

## Abstract

To enhance the surface quality of metal 3D-printed components, magnetic abrasive finishing (MAF) technology was employed for post-processing polishing. Experimental investigation employing response surface methodology was conducted to explore the impact of processing gap, rotational speed of the magnetic field, auxiliary vibration, and magnetic abrasive particle (MAP) size on the quality enhancement of internal surfaces. A regression model correlating roughness with crucial process parameters was established, followed by parameter optimization. Ultimately, the internal surface finishing of waveguides with blind cavities was achieved, and the finishing quality was comprehensively evaluated. Results indicate that under optimal process conditions, the roughness of the specimens decreased from Ra 2.5 μm to Ra 0.65 μm, reflecting a reduction rate of 74%. Following sequential rough and fine processing, the roughnesses of the cavity bottom, side wall, and convex surface inside the waveguide reduced to 0.59 μm, 0.61 μm, and 1.9 μm, respectively, from the original Ra above 12 μm. The findings of this study provide valuable technical insights into the surface finishing of metal 3D-printed components.

## 1. Introduction

Selective Laser Melting (SLM) is an advanced manufacturing method that utilizes a high-energy laser beam to selectively melt and solidify metal powder within specific regions, thereby achieving “near-net-shape” fabrication of three-dimensional complex structures [1,2,3]. Based on the principle of “layer-by-layer” deposition and stacking, this technology enables direct fabrication of solid objects from digital models, offering advantages such as design flexibility, short production cycles, and cost savings. Currently, SLM finds widespread applications in critical fields such as aerospace, biomedicine, and electronic communications [4,5,6,7]. Particularly in the radio transmission field, metal waveguides often leverage the advantages of SLM technology for manufacturing their intricate structures [8].

However, due to inherent defects such as “unmelted powder adhesion” and “balling effect”, the surface quality of SLM-formed components is often poor [9,10]. Studies have shown that [11,12] the roughness of the internal surface of waveguides significantly affects their signal transmission quality, making it necessary to polish the internal surfaces. Traditional technologies face polishing difficulties due to structural interference and poor abrasive accessibility within complex cavities. Magnetic abrasive finishing (MAF) can achieve surface finishing by controlling the force and motion of the magnetic abrasive particles (MAPs) through external magnetic fields. For non-magnetic workpieces, MAF cannot be affected by the geometric structure of the workpiece, making it applicable to polish some complex internal structures [13,14].

Currently, MAF has been extensively studied by scholars worldwide. Poudel et al. [15] conducted magnetic abrasive finishing on 3D-printed parts under various process parameters, revealing the intrinsic connection between printing parameters and the final surface quality after MAF. Barman [16] and Zou et al. [17] introduced spiral feeding mechanisms to cylindrical magnetic poles, resulting in denser abrasive trajectories and improving polishing uniformity. Guo et al. [18] applied high-frequency vibration to the magnetic poles using a voice coil motor, significantly enhancing the surface quality of microgrooves without damaging the microstructure. Zhang et al. [19] employed a vibration cylinder to drive the magnetic pole, generating linear reciprocating motion and effectively enhancing the surface integrity of 3D-printed components. Misra [20], Ghosh [21], and Kala [22] investigated the effects of factors such as workpiece material, abrasive-workpiece contact status, MAP composition, and original workpiece morphology on finishing quality in established material removal models, revealing the evolution laws of surface quality.

For the internal surface, Jiao [23] achieved the deburring of microholes using radially magnetized cylindrical magnetic poles with MAPs. Zhang [24] effectively increased the micro-cutting depth of abrasives by introducing auxiliary magnetic poles for polishing the internal surfaces of high-hardness and thick-walled round tubes. Muhamad et al. [25] proposed the electrochemical-assisted MAF technique, which improved the polishing efficiency and effect. Li [26] polished the internal surfaces of round tubes using self-developed novel viscoelastic MAPs and derived a mathematical model for the time-variable material removal rate. Sasan et al. [27] fed self-rotating pole heads along the curved contour of internal surfaces, resulting in a 70% improvement in surface quality. The literature mentioned above investigated vital process parameters of MAF from experimental or theoretical perspectives and addressed some of the challenges in internal surface finishing. However, research on the MAF of complex internal surfaces with blind cavities is scarce, as the processed objects mainly consist of flat surfaces or round tubes.

In this paper, the MAF technology was utilized to perform surface finishing on the complex internal surfaces of waveguides made by SLM. The printed components were sequentially processed with steel balls and bonded-type MAPs for rough and fine processing. Finally, a comprehensive evaluation of the uniformity and consistency of the polishing quality inside the waveguide was conducted.

## 2. Experiment

### 2.1. Implementation

**Figure 1:**
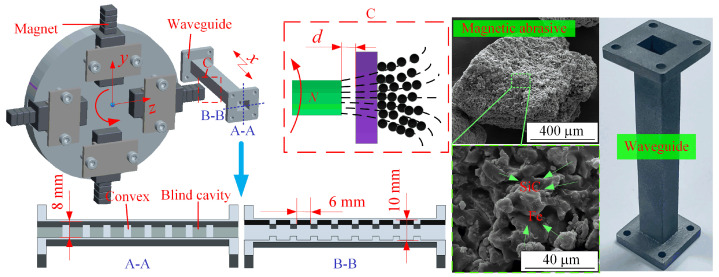
Schematic diagram of the MAF.

Figure 1 illustrates the implementation principle of MAF. The self-rotation of the magnetic assembly enables the generation of a rotating magnetic field. Within the dynamic magnetic field, MAPs contained inside the waveguide assemble into the adaptable magnetic brush, which exerts polishing pressure and sliding speed on the inner surface, inducing a micro-cutting effect. The bonded-type MAPs are composed of Fe powders (the average diameter is 21 μm), SiC powders (the average diameter is 21 μm), and the binder, which are cured by heating, with an Fe content of 67.3 wt.%. The waveguide is fed along the *x* direction while supplemented with high-frequency vibration to enhance processing efficiency and quality. The waveguide is rapidly formed using AlSi10Mg metal powders through SLM technology. Sections A-A and B-B reveal the presence of several blind cavities inside the waveguide, indicating its complex structure. These blind cavities are primarily used to regulate the information transmission mode of the waveguide, making the blind-cavity bottom crucial for its operational performance. This work focused on the experimental research of the blind cavity bottom as the primary research object. Preliminary work indicates that the processing scheme in this paper can achieve good results in finishing blind cavity structures [28].

**Figure 2:**
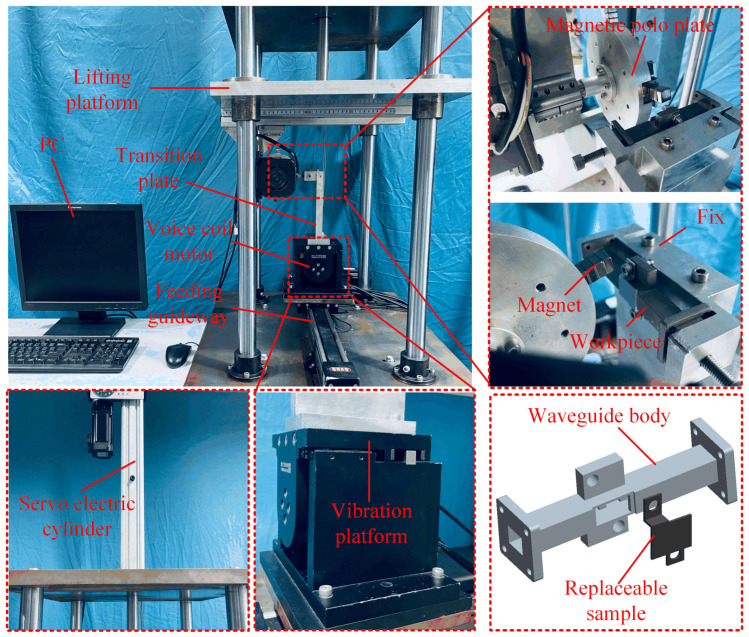
Physical picture of the experimental setup.

Figure 2 shows the physical picture of the MAF device. The stepper motor rotates the magnetic pole plate, generating the magnetic field with a gradient. The voice coil motor applies high-frequency vibration to the waveguide workpiece through the fixture. The electric servo cylinder drives the lifting platform to complete the “tool alignment” operation. The synchronous belt guide rail achieves the feeding motion of the waveguide. The magnetic-field-generating device can slide on the guide rail of the lifting table to adjust the processing clearance. To save experimental costs and facilitate the measurement of surface morphology, a split-type waveguide workpiece, consisting of the specimen and the waveguide body, is used. The PC implements the control and adjustment of all motors and drive equipment.

### 2.2. Conditions and Method

**Figure 3:**
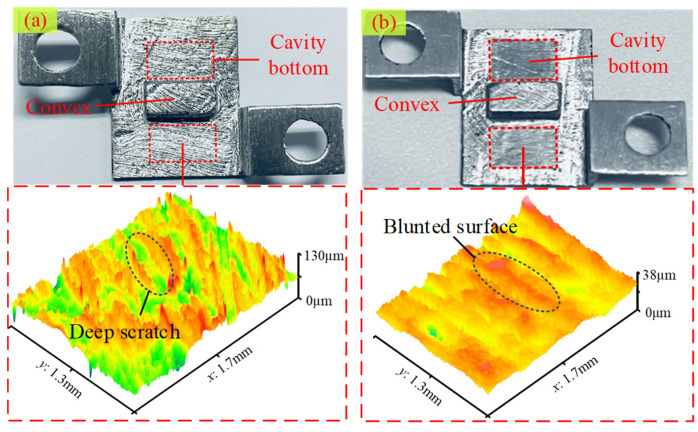
Surface morphology of the specimens. (**a**) The original morphology; (**b**) The morphology after rough polishing.

In the experiments, the surface roughness values were measured using a contact-type roughness tester (Mitutoyo, Tokyo, Japan, SJ-210). The workpieces were cleaned using an ultrasonic cleaning machine filled with anhydrous ethanol (KL-1012, Shenzhen KeLi Ultrasonic Cleaning Equipment Co., Ltd., Shenzhen, China). The amount of magnetic abrasive used for each experiment was measured using an electronic precision balance (XPR205DU, Mettler Toledo Technology (China) Co., Ltd., Shanghai, China, accuracy 0.1 mg). Surface morphology and line roughness were measured using a laser confocal microscope (UP-lambda, Aitech Instrument Technology Co., Ltd., Nanjing, China). The morphology of the magnetic abrasives was examined using a scanning electron microscope (SEM, Quanta FEG 250, Shanghai, China). Roughness values were measured at 5 evenly chosen locations within the finished surface, and the average of these measurements was computed to determine the Ra value for the area. Due to the inferior surface quality of the original printed part, 0.5 g steel balls (diameter of 0.6 mm) were initially used as magnetic abrasives for rough polishing to enhance processing efficiency. The processing clearance was set at 2 mm, and the parameters of the vibrator were adjusted to an amplitude of 0.2 mm and a frequency of 15 Hz. The feeding motion was achieved at a speed of 7 mm/s, with rough polishing lasting for 60 min [28]. Figure 3 illustrates the surface morphology of the specimen. It can be observed that there are numerous apparent scratches on the original surface, caused by the supports in the SLM process, resulting in a surface roughness of over 10 μm. Following rough polishing, the surface peaks were flattened, yet the peak-to-valley value remained relatively large, with the surface roughness reduced to around 2.5 μm.

After rough polishing, fine polishing was performed using 0.5 g of MAPs for 60 min. To examine the impact of process parameters on surface smoothness and optimize these parameters, experimental research was conducted using the response surface methodology. The surface roughness Ra was selected as the output response, with the inputs being four key factors: processing gap, magnetic pole speed, vibrational frequency, and MAP size. The specific factors and levels are shown in Table 1.

Based on the Box–Behnken design, 29 sets of experiments were conducted, and the experimental results are presented in Table 2.

## 3. Results and Discussion

### 3.1. Regression Model

To establish the functional relationship between the inputs and the response, a quadratic model was used for regression fitting. Table 3 presents the analysis of variance results for the quadratic model. The analysis reveals an “F-value” of 51.37, demonstrating high significance, as evidenced by a “Prob > F” value well below 0.05. Regarding the “lack-of-fit” term, the model demonstrates robust reliability, with a “Prob > F” value surpassing 0.05. The quadratic model exhibits consistency between the predicted R-squared and adjusted R-squared, with a difference of less than 0.2. Additionally, an “Adequacy Precision” of 26.297 indicates an abundance of signal sources, supporting the applicability of the model for guiding the variable space.

In this model, the input parameters A, B, C, and D, along with their interaction terms (AB), (AC), (AD), (BC), and (CD), as well as the second-order terms B^2^, C^2^, and D^2^, exhibit evident significance. The regression equation for roughness Ra was obtained using Expert-design software (Version 12.0.3), as shown in Equation (1).

(1) $$\begin{array}{l} \mathrm{Ra}=5.491+0.832\times A-0.015\times B-0.155\times C-7.250E^{-3}\times D+0.912E^{-3}\times A\times B\\ \;\;-0.016\times A\times C-6.50E^{-3}\times A\times D+0.115E^{-3}\times B\times C-0.21E^{-4}\times B\times D\\ \;\;+0.550E^{-3}\times C\times D-0.035\times A^{2}+8.927E^{-6}\times B^{2}\\ \;\;+2.958E^{-3}\times C^{2}+0.333E^{-3}\times D^{2} \end{array}$$

**Figure 4:**
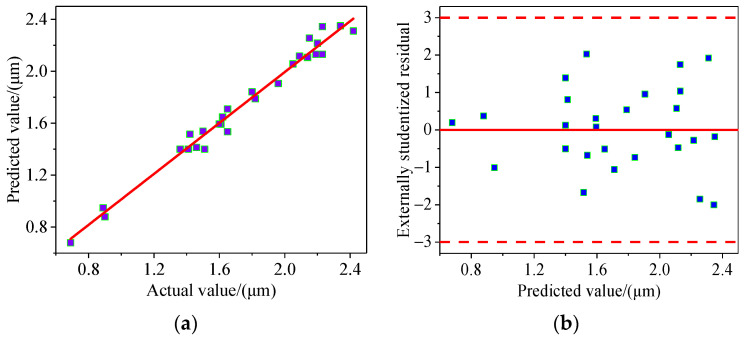
Predictive performance of the model. (**a**) Relationship between predicted and actual values; (**b**) Relationship between predicted values and residuals.

Figure 4 depicts the predictive performance of the model. The predicted values exhibit an approximately linear relationship with the experimental values. Moreover, the externally studentized residuals are randomly distributed, indicating the reliability of the dataset. Therefore, the model demonstrates high credibility for predicting roughness.

### 3.2. Separate Impacts of Linear Variables on the Response

**Figure 5:**
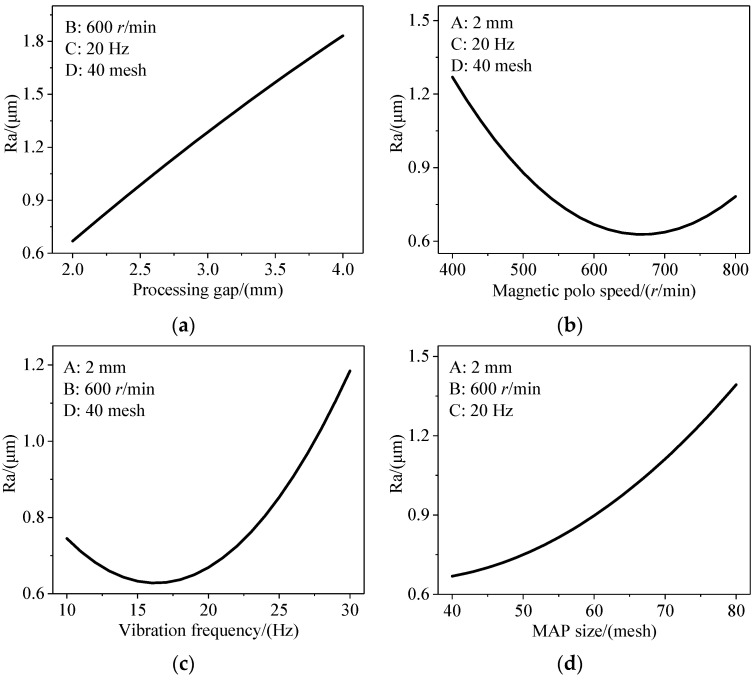
Relationship between the linear term and the surface roughness. (**a**) Ra vs. processing gap; (**b**) Ra vs. magnetic polo speed; (**c**) Ra vs. vibration frequency; (**d**) Ra vs. MAP size.

Figure 5 illustrates the effect of linear variables on Ra. From Figure 5a, it is evident that reducing the processing clearance contributes to a decrease in roughness. This phenomenon arises from the weakening of magnetic field control over the MAPs as they depart from the magnetic pole, resulting in poorer rigidity of the magnetic force brush and ultimately leading to lower processing efficiency.

From Figure 5b, it can be observed that the response curve exhibits an initial decreasing and then increasing trend. Increasing the magnetic pole rotation speed within a specific range can enhance the sliding speed and improve processing efficiency. However, when the magnetic field rotation velocity is too high, the acceleration duration of the MAPs is shortened. At this point, the MAPs are prone to breaking free from the confinement of the magnetic field force, limiting their sliding strokes and affecting the polishing quality.

Figure 5c indicates the response curve initially decreasing before rising as the vibration frequency increases. That is because increasing the vibration frequency within a specific range can make the polishing trajectory of MAPs more dense, effectively improving the processing efficiency and the uniformity of processing quality. However, due to the tiny size of the blind cavity, intense lateral vibration can exacerbate collisions between MAPs in the limited space, resulting in significant randomness and uncertainty in the sliding path, which may even adversely affect the polishing effect [29,30].

Figure 5d demonstrates a negative correlation between the particle size of MAPs and the response value. This is because smaller-sized MAPs are unable to encapsulate sufficient ferromagnetic materials and abrasives, resulting in decreased magnetic permeability and cutting performance.

### 3.3. Combined Impacts of Factors on the Response

**Figure 6:**
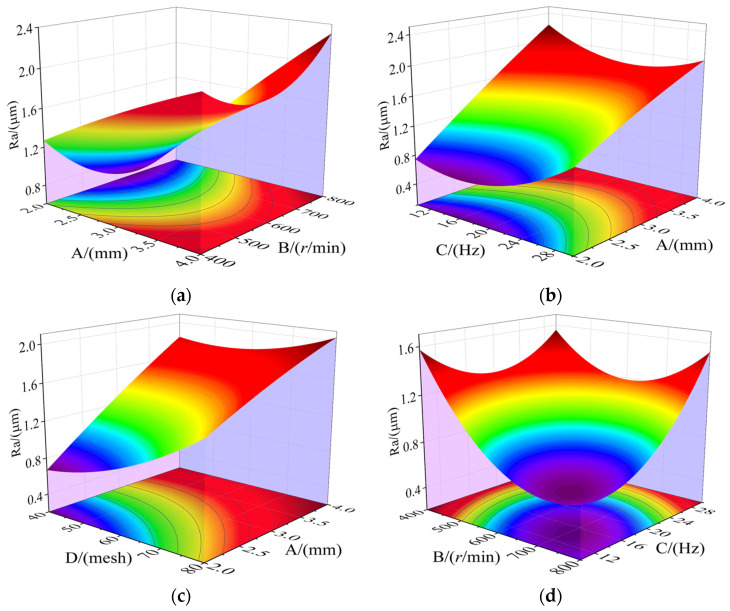
Combined impacts of items on roughness. (**a**) C = 20 Hz, D = 40 mesh; (**b**) B = 600 r/min, D = 40 mesh; (**c**) B = 600 r/min, C = 20 Hz; (**d**) A = 2 mm, D = 40 mesh.

According to the theory of analysis of variance (ANOVA), a higher F-value indicates a stronger significance. Considering this, four interaction terms with higher F-values were selected for analysis, ranked by significance as follows: (BC), (AB), (AC), and (AD). Figure 6 illustrates the effect of interaction terms on roughness. Figure 6a shows that the roughness can reach a minimum value when factor A is set to 2 mm and factor B approaches 600 r/min. Similarly, Figure 6b shows that the minimum roughness value can be achieved when factor A is set to 2 mm and factor C approaches 16 Hz. The underlying laws in both figures align with the earlier analysis. The processing clearance mainly influences the polishing pressure, while the magnetic pole rotation speed and vibration frequency primarily affect the sliding speed of MAPs. According to the Preston equation [31], increasing the normal polishing pressure and relative sliding speed can enhance the material removal rate. However, due to the confined space inside the waveguide, intense interactions exist among the MAPs and between the MAPs and the internal wall, which would be the dominant factor limiting the relative sliding speed.

It can be seen from Figure 6c that when factor A and factor D take the minimum value at the same time, the roughness reaches the minimum value. That is because the size of MAPs mainly affects the embedded depth of abrasives on the workpiece surface; that is, the larger the volume of MAPs, the greater the polishing pressure. Figure 6d reveals a compromise between magnetic pole rotation speed and vibration frequency within a specific range, which allows for achieving the optimal polishing effect. This phenomenon is consistent with the above research results. In a restricted space, there would be limitations in increasing the relative motion speed.

### 3.4. Process Optimization

The numerical algorithm embedded in the Design-Expert software was utilized to obtain the best combination of process parameters. The process parameters need to be rounded, and experimental validation was conducted considering the actual operating conditions. Detailed results are displayed in Table 4, revealing the rounded optimal process conditions as follows: processing clearance of 2 mm, magnetic pole speed of 650 r/min, vibration frequency of 16 Hz, and MAP size of 40 mesh. Under the optimal process parameters, a roughness of Ra 0.65 μm can be obtained, with an error of 7.7% between theoretical and experimental values, meeting the requirement of engineering practice and confirming the rationality of the regression model.

**Figure 7:**
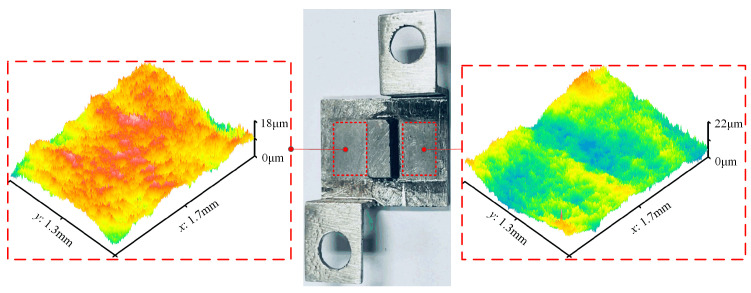
The polishing effect of the samples under the optimal parameters.

Figure 7 illustrates the polishing effect of the specimen under the optimal parameters. It can be observed that the surface becomes smoother after MAF, and there is a significant improvement in the three-dimensional morphology. And the Vickers hardness of samples increases from the original 120 HV to 220 HV after processing.

### 3.5. Comprehensive Evaluation

**Figure 8:**
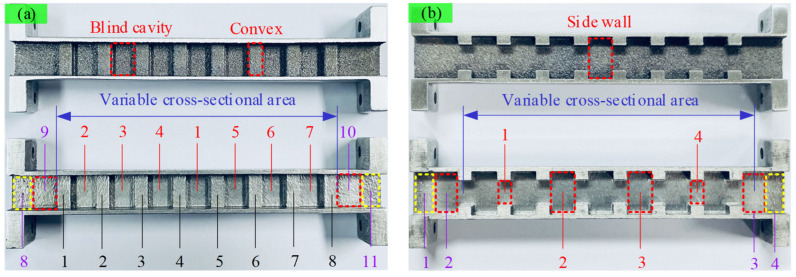
Polishing effect on the internal surface of the waveguide. (**a**) Section A-A; (**b**) Section B-B.

The internal surface of the waveguide was sequentially polished using steel balls and MAPs. The processing effect is illustrated in Figure 8. It can be seen that the internal surface before polishing is uneven and lacks metallic luster. In contrast, the bottoms and sides of the blind cavities become remarkably smooth after polishing. Additionally, there is a certain improvement in the quality of the convex surfaces.

(1) Surface morphology before processing

**Figure 9:**
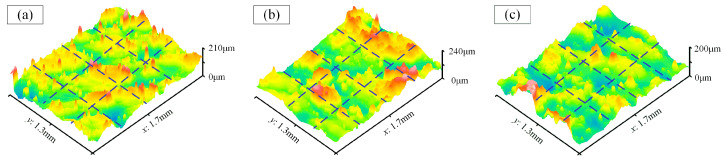
The three-dimensional morphology before processing. (**a**) Cavity bottom; (**b**) Convex surface; (**c**) Side wall.

Figure 9 shows the three-dimensional morphology on the unpolished internal surface. Five evenly spaced lines were selected on the morphology map (as indicated by the blue dashed lines), and the morphology data were extracted from each line. The roughness values were calculated using the post-processing software Gwyddion (Version-2.65), and their average value was taken as the Ra value for that region. It can be observed that there are numerous peaks and valleys on the original internal surface, with a significant peak-to-valley difference, resulting in an average roughness of approximately Ra 12 μm.

(2) Morphology on the cavity bottom after processing

**Figure 10:**
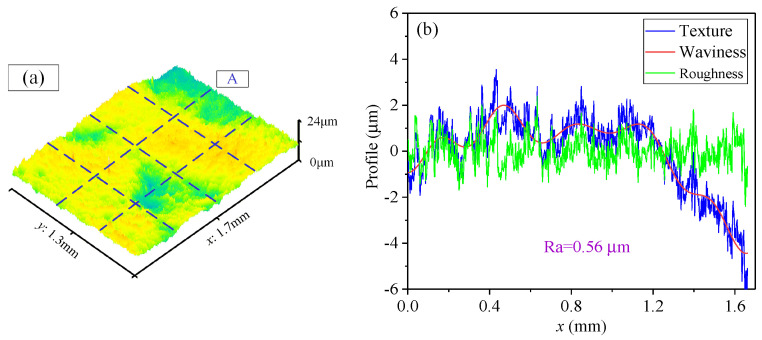
The surface morphology of the cavity bottom (Number 1). (**a**) Three-dimensional morphology; (**b**) Two-dimensional profile.

Figure 10 depicts the surface morphology on the cavity bottom (Number 1) within the variable cross-section region. It can be known that the surface morphology has been effectively improved after MAF. The profile characteristics from a measurement line A shown in the box of Figure 10a were analyzed, as shown in Figure 10b. From the waviness curve, there is some fluctuation in the polished bottom of the cavity, but its surface microtopography has been significantly enhanced. The original surface of 3D-printed parts is extremely rough, especially with spherical particles and metal powder adhesion, resulting in significant waviness and a peak-to-valley height of up to 200 μm. After magnetic abrasive finishing, the peak-to-valley difference on the surface is reduced to around 20 μm, leading to a significant improvement in surface waviness. However, further reducing the peak-to-valley difference is limited due to the effects of work hardening and surface morphology size effects. Finally, the roughness Ra has been reduced to 0.56 μm.

**Figure 11:**
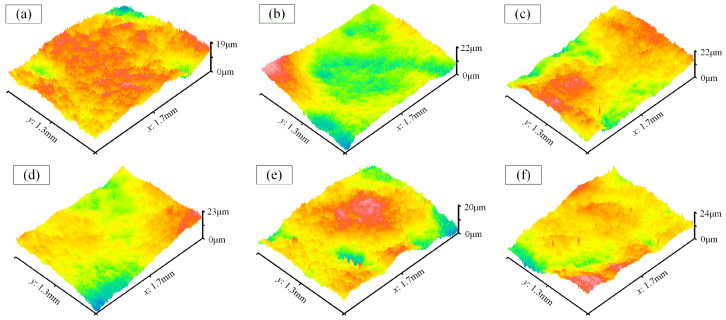
The surface morphology of the cavity bottom. (**a**–**f**) Number 2–7.

Figure 11 shows the surface morphology of the cavity bottom (Number 2–7). Among them, the maximum and minimum Ra values are 0.62 μm and 0.56 μm, respectively, with an average of 0.59 μm. Additionally, they exhibit similar peak-to-valley values, indicating good uniformity in the surface quality.

**Figure 12:**
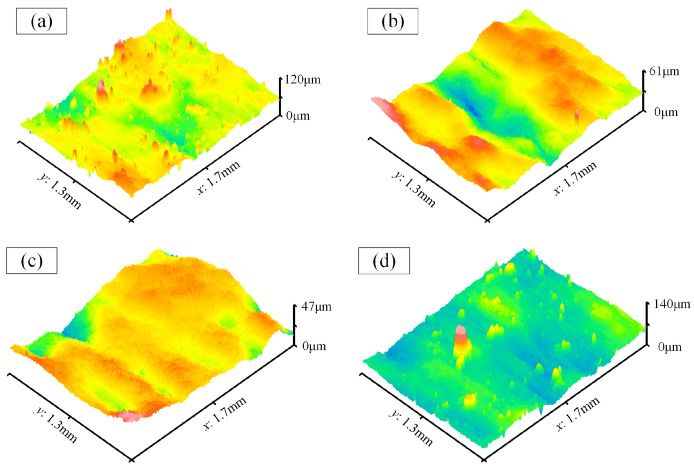
The surface morphology on the cavity bottoms at both ends of the waveguide. (**a**–**d**) Number 8–11.

Figure 12 displays the surface morphology on the cavity bottoms at both ends of the waveguide, revealing a notable deficiency in polishing effect. This deficiency primarily stems from the interference posed by the flange structures situated at both ends of the waveguide, which disrupt the feeding motion of the magnetic pole plate. Consequently, the magnet encounters difficulty aligning directly with the region beneath the flange, resulting in minimal to negligible polishing activity within this specific area. As denoted by numbers 8 and 11, the peak-to-valley values exhibit significant prominence. In proximity to the flange (designated as numbers 9 or 10), inadequate processing ensues owing to the abbreviated dwell time of the magnet in this zone.

(3) Morphology on the convex surface after processing

**Figure 13:**
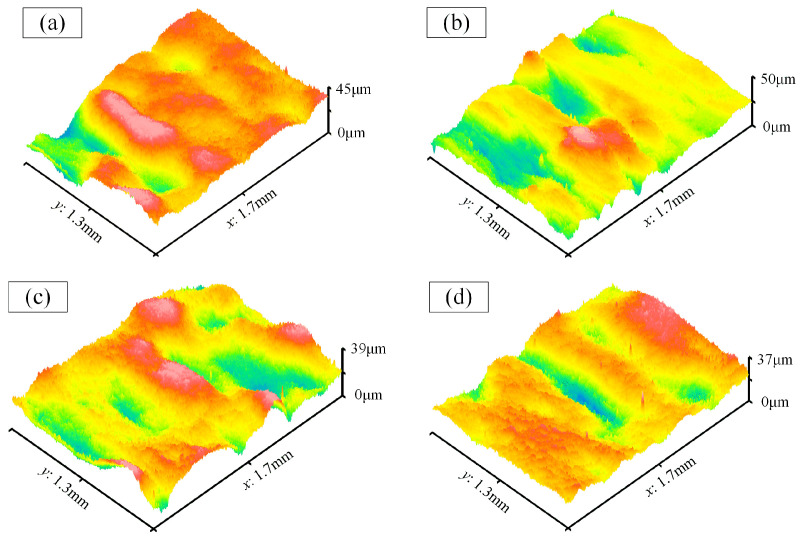
The morphology on the convex surface after processing. (**a**) Label-2; (**b**) Label-4; (**c**) Label-6; (**d**) Label-8.

Figure 8a demonstrates that many large adhered particles and unmelted powders on the original convex surface have been effectively removed, yet surface irregularities persist. The partial three-dimensional morphology images of the convex surfaces were extracted for evaluation, and the results, depicted in Figure 13, reveal the absence of sharp peaks on convex surfaces, replaced by lower peaks and smoother surfaces. In this region, the effectiveness of the magnetic field diminishes as the convex surface lies farther from the magnetic field source compared to the cavity bottom. Measurements indicate that the average Ra value of the convex surfaces is approximately 1.9 μm.

(4) Morphology on the side wall after processing

**Figure 14:**
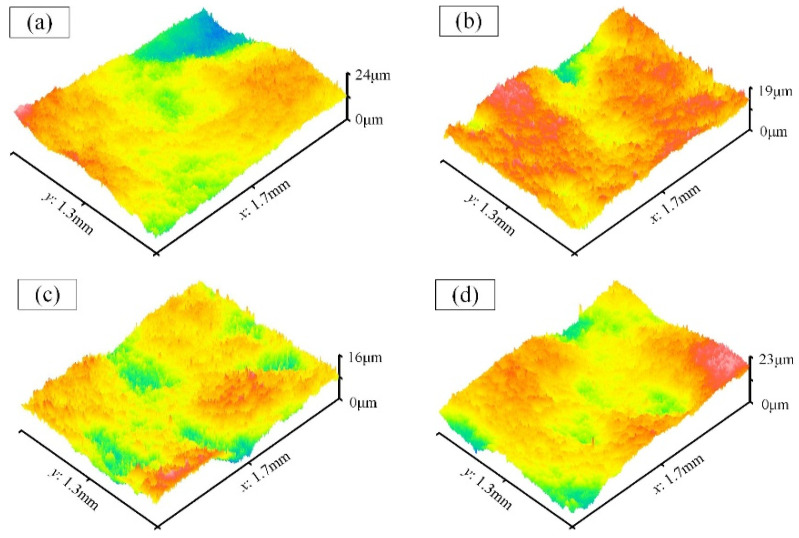
The morphology images on the side walls within the variable cross-section region. (**a**) Label-1; (**b**) Label-2; (**c**) Label-3; (**d**) Label-4.

The partial three-dimensional morphology images of the side walls within the variable cross-section region were extracted for analysis, as depicted in Figure 14. From those, it is evident that the asperities on these surfaces have significantly decreased. Measurements indicate that the average Ra value of the side walls is approximately 0.61 μm.

**Figure 15:**
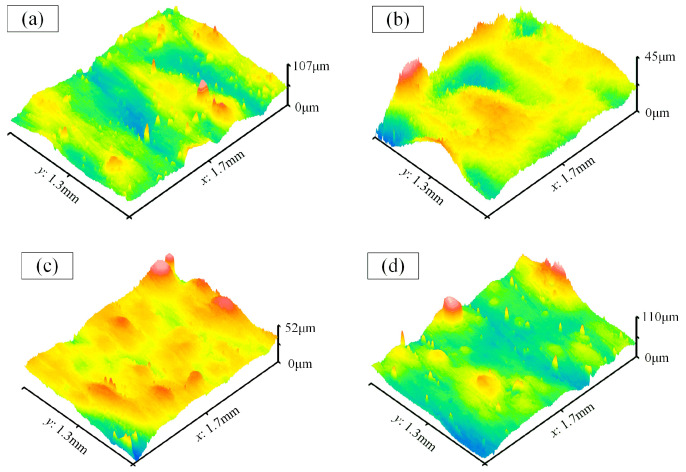
The morphology images on the side walls at both ends of the waveguide. (**a**) Label-1; (**b**) Label-2; (**c**) Label-3; (**d**) Label-4.

Figure 15 illustrates the morphology images on the side walls at both ends of the waveguide. It can be observed that the polishing effects in those areas are unsatisfactory. This is also attributed to interference caused by the flange structures. Particularly, the regions labeled as 1 and 4 at the ends received almost no polishing.

## 4. Conclusions

The present study sequentially employed steel balls and MAPs for polishing the internal surface of the waveguide. The inherent correlation between the process parameters and the internal surface quality was established and a comprehensive evaluation of the microscopic morphology of different regions inside the waveguide was carried out. The primary research findings are as follows:

(1) Utilizing steel balls as magnetic abrasives for rough processing can facilitate a rapid decrease in internal surface roughness.
(2) When using magnetic abrasive particles as polishing tools, the optimal process parameters include a processing gap of 2 mm, magnetic pole rotation velocity of 650 r/min, vibration frequency of 16 Hz, and MAPs size of 40 mesh.
(3) Following sequential rough and fine processing of the internal cavity of the waveguide, the Ra of the blind cavity bottom, side wall, and convex surface can be reduced to 0.59 μm, 0.61 μm, and 1.9 μm, respectively, effectively improving the surface quality inside the waveguide.

The current challenge lies in the complex task of polishing the internal surfaces. It is difficult to effectively polish the internal surface of this type of waveguide with traditional processes, which becomes a problem. Our research aims to explore a viable solution for initiating a process from being non-polishable to becoming feasible and to provide theoretical guidance and technical support for the magnetic abrasive finishing of complex internal surface structures. In the preliminary stage, the goal is to reduce the surface roughness Ra of the blind-cavity bottom to less than 1 μm without deteriorating surface accuracy. In future work, in view of the shortcomings of this work, such as dimensional accuracy, surface waviness and limited processing conditions in some areas, the author will further enhance processing quality by optimizing the performance of magnetic abrasives and introducing new techniques.

## Figures and Tables

**Table 1:** Process parameters and their levels.

Process Parameters	Levels		
	−1	0	1
A: Processing gap [mm]	2	3	4
B: Magnetic polo speed [r/min]	400	600	800
C: Vibration frequency [Hz]	10	20	30
D: MAP size [Mesh]	40 (20–40)	60 (40–60)	80 (60–80)

**Table 2:** Experimental parameters and responses.

Exp. No.	Factors and Their Levels				Ra [μm]
	A [mm]	B [r/min]	C [Hz]	D [Mesh]	
1	4	600	10	60	2.14
2	2	400	20	60	1.61
3	3	800	20	80	1.96
4	2	600	20	40	0.69
5	3	800	20	40	1.6
6	4	400	20	60	2.19
7	3	400	20	40	1.65
8	4	600	20	40	1.8
9	3	600	10	80	1.82
10	3	600	20	60	1.51
11	3	400	10	60	2.15
12	3	600	30	40	1.62
13	3	600	20	60	1.41
14	2	600	30	60	1.5
15	2	600	10	60	0.9
16	4	600	20	80	2.05
17	3	800	10	60	1.42
18	3	600	20	60	1.36
19	3	400	20	80	2.34
20	3	600	20	60	1.36
21	3	400	30	60	2.23
22	4	800	20	60	2.2
23	3	800	30	60	2.42
24	2	600	20	80	1.46
25	3	600	30	80	2.23
26	4	600	30	60	2.09
27	3	600	20	60	1.36
28	2	800	20	60	0.89
29	3	600	10	40	1.65

**Table 3:** The ANOVA for the quadratic model.

Source	SOS	DF	MS	F-Value	Prob > F
Model	5.620	14	0.401	51.370	<0.0001
A	2.450	1	2.450	313.35	<0.0001
B	0.235	1	0.235	30.110	<0.0001
C	0.337	1	0.337	43.090	<0.0001
D	0.677	1	0.677	86.640	<0.0001
AB	0.133	1	0.133	17.052	0.001
AC	0.106	1	0.106	13.520	0.002
AD	0.068	1	0.068	8.653	0.011
BC	0.211	1	0.211	27.085	<0.0001
BD	0.027	1	0.027	3.485	0.083
CD	0.048	1	0.048	6.195	0.026
A^2^	0.008	1	0.008	1.041	0.325
B^2^	0.827	1	0.827	105.867	<0.0001
C^2^	0.568	1	0.568	72.663	<0.0001
D^2^	0.115	1	0.115	14.760	0.002
Residual	0.109	14	0.008		
LOF	0.092	10	0.009	2.17	0.236
PE	0.017	4	0.004		
Sum	5.730	28			
Std. Dev.	R^2^	Adjusted R^2^	Predicted R^2^	Adequacy Precision	
0.088	0.981	0.962	0.903	26.297	

**Table 4:** The optimized results and experimental verification.

Type	Factors				Ra (μm)		Error (%)
	A (mm)	B (r/min)	C (Hz)	D (Mesh)	Predicted	Experimental	
Optimized	2.02	654.78	16.26	40.53	0.6	0.65	7.7
Rounded	2	650	16	40			

## Data Availability

Data are contained within the article.

## References

[1] Balanovsky A.E., Gozbenko V.E., Kargapoltsev S.K., Karlina A.I., Karlina Y.I. (2020). Evaluation of influence of technological parameters on width of strengthened layer in plasma surface hardening of structural steels. IOP Conf. Ser. Mater. Sci. Eng..

[2] Ucak N., Cicek A., Aslantas K. (2022). Machinability of 3D printed metallic materials fabricated by selective laser melting and electron beam melting: A review. J. Manuf. Process..

[3] Karlina A.I., Karlina Y.I., Kondratiev V.V., Kononenko R.V., Breki A.D. (2023). Study of wear of an alloyed layer with chromium carbide particles after plasma melting. Crystals.

[4] Presotto A.G.C., Cordeiro J.M., Presotto J.G.C., Rangel E.C., Da Cruz N.C., Landers R., Barao V.A.R., Mesquita M.F. (2021). Feasibility of 3D printed Co–Cr alloy for dental prostheses applications. J. Alloys Compd..

[5] Ponnusamy P., Rahman Rashid R.A., Masood S.H., Ruan D., Palanisamy S. (2020). Mechanical properties of SLM-printed aluminium alloys: A review. Materials.

[6] Baciu E.R., Cimpoesu R., Vitalariu A., Baciu C., Cimpoesu N., Sodor A., Zegan G., Murariu A. (2020). Surface analysis of 3D (SLM) Co–Cr–W dental metallic materials. Appl. Sci..

[7] Rasiya G., Shukla A., Saran K. (2021). Additive manufacturing: A review. Mater. Today Proc..

[8] Kuhling J., Dahle R., Chowdhry D., Laforge P. (2020). Applying additive manufacturing to integrate coaxial connectors with 3D printed waveguides for cascaded RF link applications. Addit. Manuf..

[9] Lindstrom V., Lupo G., Yang J., Turlo V., Leinenbach C. (2023). A simple scaling model for balling defect formation during laser powder bed fusion. Addit. Manuf..

[10] Tyagi P., Goulet T., Riso C., Stephenson R., Chuenprateep N., Schlitzer J., Benton C., Garcia-Moreno F. (2019). Reducing the roughness of internal surface of an additive manufacturing produced 316 steel component by chempolishing and electropolishing. Addit. Manuf..

[11] Salazar R., Pizarro F., Vasquez D., Rajo-Iglesias E. (2022). Assessment of 3D-printed waveguides using conductive filaments and a chloroform-based smoothing process. Addit. Manuf..

[12] Almeshehe M., Murad N., Rahim M., Ayop O., Samsuri N., Abd Aziz M., Osman M. (2022). Surface roughness impact on the performance of the 3D metal printed waveguide coupler at millimeterwave band. Eng. Sci. Technol. Int. J..

[13] Babbar R., Misra A. (2023). Development of a novel magnetic abrasive finishing setup with replenishment of abrasive particles from external source and its experimental investigations. CIRP J. Manuf. Sci. Technol..

[14] Kaushik A., Singh P., Kumar H., Singh L. (2023). Experimental comparison of unbonded, agglutinated and sintered SiC-based magnetic abrasive particles in magnetic abrasive finishing process. J. Magn. Magn. Mater..

[15] Poudel B., Nguyen H.X., Kwon P., Chung H. (2023). Selective laser melting of oxide dispersion strengthened MA956 alloy and its surface finishing by magnetic field assisted finishing. J. Manuf. Process..

[16] Barman A., Das M. (2017). Toolpath generation and finishing of bio-titanium alloy using novel polishing tool in MFAF process. Int. J. Adv. Manuf. Technol..

[17] Zou Y.H., Xie H.J., Zhang Y.L. (2020). Study on surface quality improvement of the plane magnetic abrasive finishing process. Int. J. Adv. Manuf. Technol..

[18] Guo J., Feng W.H., Jong H.J.H., Suzuki H., Kang R.K. (2020). Finishing of rectangular microfeatures by localized vibration-assisted magnetic abrasive polishing method. J. Manuf. Process..

[19] Zhang J., Chaudhari A., Wang H. (2019). Surface quality and material removal in magnetic abrasive finishing of selective laser melted 316L stainless steel. J. Manuf. Process..

[20] Misra A., Pandey P.M., Dixit U.S. (2017). Modeling and simulation of surface roughness in ultrasonic assisted magnetic abrasive finishing process. Int. J. Mech. Sci..

[21] Ghosh G., Sidpara A., Bandyopadhyay P.P. (2021). Experimental and theoretical investigation into surface roughness and residual stress in magnetorheological finishing of OFHC copper. J. Mater. Process. Technol..

[22] Kala P., Sharma V., Pandey P.M. (2017). Surface roughness modelling for double disk magnetic abrasive finishing process. J. Manuf. Process..

[23] Jiao A.Y., Zhang G.F., Liu B.H., Liu W.J. (2020). Study on improving hole quality of 7075 aluminum alloy based on magnetic abrasive finishing. Adv. Mech. Eng..

[24] Zhang J., Hu J.L., Wang H., Kumar A.S., Chaudhari A. (2018). A novel magnetically driven polishing technique for internal surface finishing. Precis. Eng..

[25] Muhamad M.R., Zou Y., Sugiyama H. (2016). Investigation of the finishing characteristics in an internal tube finishing process by magnetic abrasive finishing combined with electrolysis. Trans. IMF.

[26] Li W.H., Li X.H., Yang S.Q., Li W.D. (2018). A newly developed media for magnetic abrasive finishing process: Material removal behavior and finishing performance. J. Mater. Process. Technol..

[27] Sasan K.A., Mosaddegh P., Alireza F.T. (2017). Study on magnetic abrasive finishing of spiral grooves inside of aluminum cylinders. Int. J. Adv. Manuf. Technol..

[28] Wang L.Y., Sun Y.L., Chen F.Y., Zhang G.G., Zhang P., Zuo D.W. (2022). Experimental study on vibration-assisted magnetic abrasive finishing for internal blind cavity by bias external rotating magnetic pole. Precis. Eng..

[29] Grover V., Singh A.K. (2018). Modelling of surface roughness in a new magnetorheological honing process for internal finishing of cylindrical workpieces. Int. J. Mech. Sci..

[30] Arora K., Singh A.K. (2021). Theoretical and experimental investigation on surface roughness of straight bevel gears using a novel magnetorheological finishing process. Wear.

[31] Deja M. (2023). The use of Preston equation to determine material removal during lap-grinding with electroplated CBN tools. Wear.

